# 

**Published:** 1867-12

**Authors:** 


### CONTENTS.

### ORIGINAL CONTRIBUTIONS.

ART. XLIX. WHY DO WE GIVE ALCOHOLIC STIMULANTS IN SHOCK? By D. W. YOUNG, M.D., Aurora, Ill., ... 705

ART. L. TRIAL OF TETRACHLORIDE OF CARBON AS AN ANÆSTHETIC—DANGEROUS EFFECTS. By E. ANDREWS, M.D., Professor of Principles and Practice of Surgery, Chicago Medical College, ... 720

ART. LI. A NEW ECRASEUR FORCEPS. By E. ANDREWS, M.D., Prof. of Principles and Practice of Surgery, Chicago Medical College, ... 722

ART. LII. ON THE SOCIAL EVILS, WITH A PLAN FOR THEIR DIMINUTION AND A PLEA FOR THE INNOCENT AND HELPLESS. By GEORGE J. ZEIGLER, M.D., ... 724

### SELECTIONS.

Clinical Papers on Ear Disease. By D. B. St. John Roosa, M.D., Prof. in the University Medical College, ... 730

Delirium Tremens successfully treated with Coffee. By W. R. Whitehead, M.D., New York, ... 733

Action of Belladonna in Disease of the Cornea. By Joseph S Hildreth, M.D., Chicago, ... 736

Weimar Cholera Conference, ... 740

### BOOK NOTICES.

The Practice of Medic ne and Surger applied to the Diseases and Accidents Incident to Women. By William H. Byford, ... 746

Studies in Pathology and Therapeutics. By Samuel Henry Dickson, ... 747

Headaches: Their Causes and their Cure. By Henry G. Wright, ... 747

Inhalation: Its Therapeutics and Practice. By J. Solis Cohen, ... 747

Notes on the Origin, Nature, Prevention, and Treatment of Asiatic Cholera. By John C. Peters, ... 747

Epidemic Meningitis, or Cerebro-Spinal Meningitis. By Alfred Stille, ... 747

Hufeland's Art of Prolonging Life. Edited by Erasmus Wilson, ... 747

Biennial Retrospect of Medicine, Surgery, and Allied Sciences. Edited by Mr. H. Power, Dr. Anstie, Mr. Holmes, Mr. Thomas Windsor, etc., ... 747

Lectures on Diseases of Women. By Charles West, ... 748

### EDITORIAL.

Close of the Volume, ... 748

Transactions of the Illinois State Medical Society, ... 748

Professional Appointments, ... 748

Death of James F. Spain, M.D., Urbana, Ohio, ... 748

Report on the Sanitary Condition and Prevalence of Disease in Chicago during the Months of July, August, and September, 1867, ... 749

The Half-Yearly Compendium of Medical Science, ... 757

Money Receipts, ... 758

Mortality Report for the Month of October, ... 759

Chestnut Leaves in Pertussis, ... 760

Winter Retreat for Consumptives, ... 760

